# Re-evaluating the occupation history of Koh Ker, Cambodia, during the Angkor period: A palaeo-ecological approach

**DOI:** 10.1371/journal.pone.0203962

**Published:** 2018-10-10

**Authors:** Tegan Hall, Dan Penny, Rebecca Hamilton

**Affiliations:** 1 School of Geosciences, The University of Sydney, Sydney, NSW, Australia; 2 School of Culture, History and Language, The Australian National University, Canberra, ACT, Australia; University of Otago, NEW ZEALAND

## Abstract

Throughout the Angkor period (9th to 15th centuries CE), the Khmer kingdom maintained a series of interconnected cities and smaller settlements across its territory on mainland Southeast Asia. One such city was Koh Ker, which for a brief period in the 10th century CE even served as a royal capital. The complexity of the political landscape meant the Khmer kings and the elite were particularly mobile through the Angkor period, and rupture in royal houses was common. However, while the historical record chronicles the 10th century migration of the royal seat from Koh Ker back to Angkor, the fate of Koh Ker’s domestic population has remained unknown. In this article, we reconstruct the settlement history of Koh Ker, using palaeoecological and geoarchaeological techniques, and show that human activity and land use persisted in the city for several centuries beyond the city’s abandonment by the royal court. These results highlight the utility of multi-proxy environmental reconstructions of Khmer urban settlements for re-evaluating prevailing assumptions regarding the use and occupation of Angkor-period cities.

## Introduction

**Fig 1:**
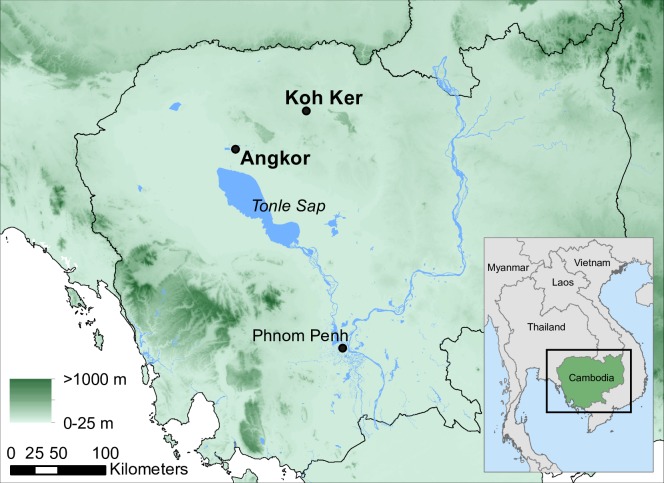
Location map. Map of Cambodia indicating the location of Koh Ker in relation to Angkor, near modern-day Siem Reap. Shading represents geographic relief. Georelief data obtained from [12], and originally sourced from [13], watercourse (blue lines) data obtained from Open Street Map (http://www.openstreetmap.org).

The city of Koh Ker was an important secondary city within the Khmer kingdom during the Angkor period (9th-15th centuries CE). Located 80 km northeast of the primate city of Angkor (Fig 1), along the royal road network that connected Angkor to many of its various peripheral settlements [1, 2], Koh Ker maintains one of the most intriguing histories of any settlement within the Khmer city network. The original survey of Aymonier [3], later enhanced by Lajonquiere [4] and Parmentier [5], reveals an expansive city housing a large reservoir (the ‘Rahal’) and several impressive temple monuments (including the grandiose stepped pyramid associated with Prasat Thom, known as the Prang), which speaks to Koh Ker’s opulence and its position within a kingdom of growing prosperity [6]. The conventional narrative describes King Jayavarman IV selecting Koh Ker as either the main (or an alternative) capital for the kingdom in 921/922 CE [7–9], until the onset of the reign of Rajendravarman in 928 CE, when the king and his court returned to the cities of Angkor. Thus ended the fleeting tenure of Koh Ker’s royal occupation [5, 10, 11] and instigated the presumed wholesale abandonment of the city and its surrounds.

The history of Koh Ker prior to and after the supposed twenty-year royal occupation is, however, poorly understood. While the consistent architectural style of the temple city’s monuments place their construction to the 10th century CE [11], a lack of unequivocal evidence for the foundation of Koh Ker by Jayavarman IV suggests that the city may have a more complex origin. Most of the inscriptions discovered within the city complex are either undated or post-date Jayavarman IV’s consecration and in fact post-date 928 AD and the migration of the capital back to Angkor [11]. Furthermore, a 2006–2007 field campaign conducted by the APSARA National Authority revealed that the baray had been modified on a recurring basis and that isolated periods of habitation in the central Koh Ker city region may have been occurring since at least the pre-Angkor period [14]. LiDAR surveys have additionally uncovered a landscape overlain with abundant ancient rice-field features throughout natural topographic depressions, as well as numerous concentric sites accompanied by radial agricultural systems, suggesting that the settlement history of the Koh Ker region may in fact extend back to the prehistoric period [15].

Several lines of evidence also cast doubt on the supposed 10th century abandonment of Koh Ker. First, an architectural reappraisal of the hospital Prasat Andon Kuk suggests that its construction occurred during the reign of Jayavarman VII, nearly two centuries beyond the capital’s relocation [11]. Also, two inscriptions (K. 674 from Prasat Dan and K. 682 from Prasat Thom) mention post-10th century development occurring at the site [7]. Surface concentrations of ceramics and stoneware, indicative of intensive 12th and 13th century occupation (and possibly into the 19th century), have been uncovered throughout the city [14, 16–19]. Moreover, evidence implying that temple and infrastructure construction occurred over multiple stages, and thus likely beyond the supposed two-decade period of occupation, come from early surveys of the site undertaken by Lajonquiere [4], which identified two separate groups of temples based on orientation. Evans [20] also highlights the impossibility of stylistically dating the vast majority of building remains beyond a broad identification as ‘Angkor-period’, given their generic, unadorned stonework. Recent differences in building material characteristics measured by Uchida et al. [21] likewise suggest that the development of the site occurred over more than one distinct phase. These assessments, however, lack the detail necessary to distinguish the timing of, or continuity between, each construction and occupation sequence, or the extent of occupation and land use that accompanied this city development.

This paper aims to test the presumption that Koh Ker was an ephemeral, single-period city. Using palaeoecological and geoarchaeological techniques, we re-evaluate the settlement and land use history of the site. Vegetation and fire regimes proximal to sites of deposition are highly responsive to human activity and can thus identify human activity and the changing nature of land use practices over time. Palaeoecological or geoarchaeological analyses of several pre-Angkor and Angkor-period cities, including Angkor Borei [22], Roluos [23], Mahendraparvata [24] and Preah Khan of Kompong Svay [25], have successfully augmented or reinterpreted occupation histories based on material cultural remains, remotely-sensed data, or art-historical and epigraphic records. The urban form of Khmer settlements are characteristically complex mosaics of urban and agricultural land uses [26–28], and thus reconstructing settlement histories here requires a multi-proxy, multidisciplinary approach.

## Regional setting

Koh Ker is situated on the extensive sedimentary basin of northern and central Cambodia [29]. The local geology largely comprises loosely consolidated Jurassic sandstones [30], while isolated limestone and rhyolitic tuff deposits, and several laterite outcrops, exist in the region closely surrounding the city [31]. Topography varies little, with elevations ranging from 50–160 m a.s.l. The climate is characterised by distinct wet and dry seasons, with approximately 90% of annual totals (1400–2000 mm) falling between May and October [32]. Due to the dynamic nature of rain-bearing systems across mainland Southeast Asia, a high level of inter-annual and inter-decadal variability is also a prominent feature of Koh Ker’s climate [33–35]. The vegetation surrounding the city today consists mainly of dry deciduous forest and woodlands, dominated by Dipterocarpaceae, riparian forest and small patches of mixed evergreen forest [36]. Numerous rice paddy fields can be found in the surrounding landscape, however many remain fallow and have transitioned to grasslands.

## Materials and methods

For this study we utilise sediment archives preserved within depositional basins throughout Koh Ker as records of long-term change in the surrounding urban landscape. The city’s main reservoir–the baray (Rahal)–is currently dry and appears to have been for some time, meaning the sediment within the reservoir is disturbed and oxidised. Three permanently wet basins were selected for coring; the moat of the main central temple, Prasat Thom, a possible inactive quarry site (Trapeang Khnar), and a small reservoir bordered on all sides by sandstone steps (Andong Preng), which was possibly associated with the Royal Palace (see [20]). The moat of Prasat Thom is approximately 32 m wide and surrounds the temple enclosure, giving a surface area of approximately 11,669 m^2^. At the time of coring, the moat had been colonised by floating vegetation (predominantly *Eichhornia crassipes* and *Pistia stratiotes*), with the exception of the southeastern quadrant, which supported a thick floating mat of grasses, ferns and aquatic plants. This suggests that colonising vegetation has been manually removed from much of the moat in the recent past. Trapeang Khnar and Andong Preng have surface areas of approximately 5200 m^2^ and 1600 m^2^ respectively. Trapeang Khnar was very heavily overgrown with a floating mat of mature swamp vegetation with fringing mature swamp forest, while Andong Preng had been cleared of a heavy Eichornia vegetation mat in the days prior to coring.

**Fig 2:**
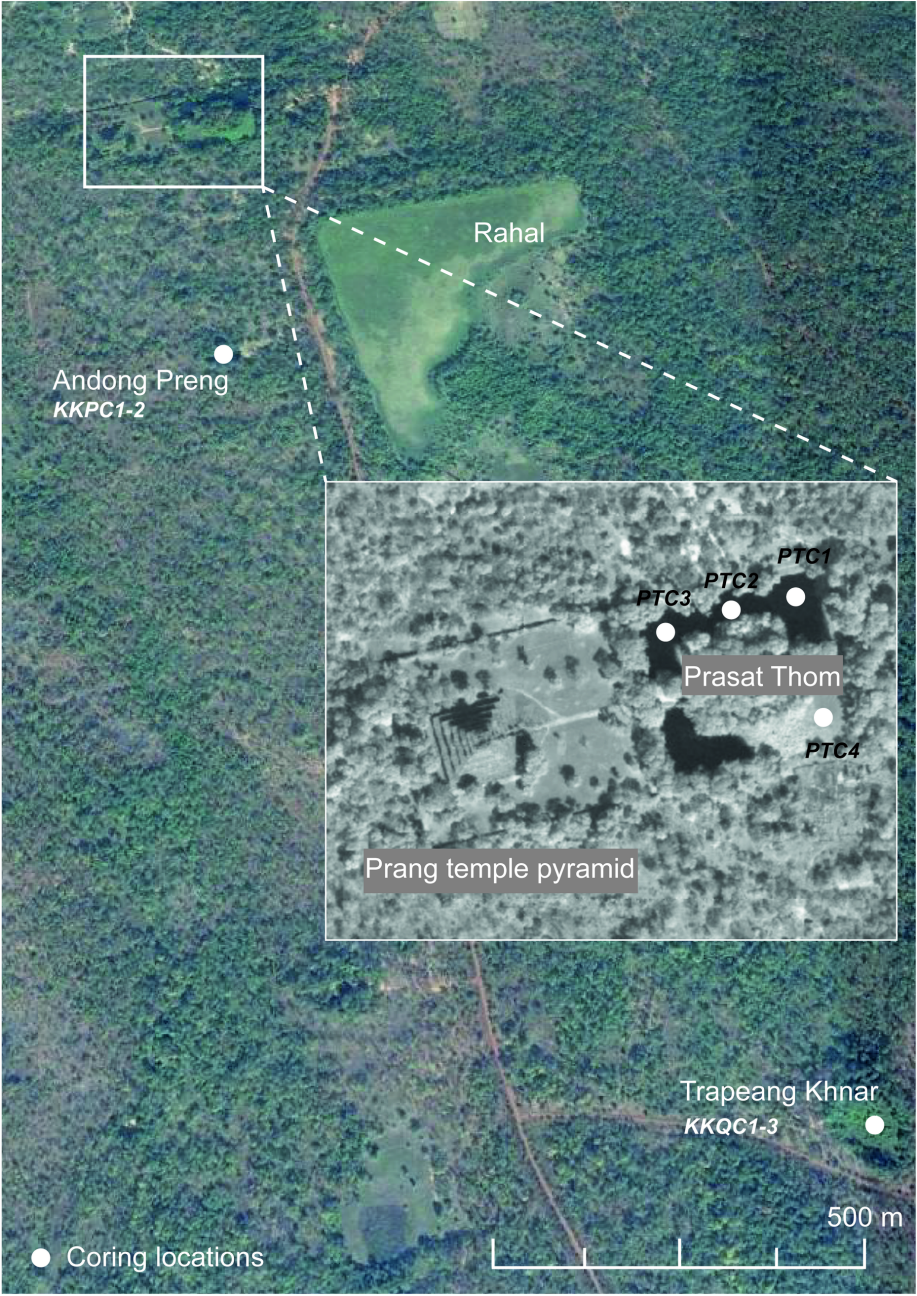
Coring locations across Koh Ker and its surrounds. The three sites Prasat Thom, Andong Preang and Trapeang Khnar and the locations from which each core was retrieved. Background image supplied by Google Earth.

We acquired all necessary permits from the Cambodian APSARA Authority for the described study, which complied with all relevant regulation. Sediment cores were obtained using a rope-operated percussion corer (see [37, 38]). The coring equipment was tethered to a rigid aluminium A-frame positioned on a floating platform, and the corer was lowered through a ‘moon-pool’ in the centre of the craft. However, in those cases where a thick floating vegetation mat precluded movement of the coring platform onto the site, the piston corer was instead carried into position and deployed by hand through a hole cut through the vegetation mat. GPS coordinates of the sampling locations were captured using a Trimble Nomad receiver using ArcPad 10.0. In total, four cores were taken from the moat of Prasat Thom, three cores (taken from the same location to capture the entire depth sequence) were taken from Trapeang Khnar, and two cores from Andong Preng (see Fig 2 for coring locations). Two cores–PTC3 from Prasat Thom and KKPC2 from Andong Preng–were selected for further detailed analysis as these archives presented the best temporal coverage and resolution (see chronology results below). Cores were cut into roughly one-metre lengths before being shipped to the University of Sydney.

Cores PTC3 and KKPC2 were split longitudinally in the laboratory, logged and photographed. Logging protocol followed Schnurrenberger et al. [39], using Munsell colour charts to define the hue, value (darkness) and chroma (strength) of the sediment colour. Magnetic susceptibility of one half of the split-core was measured using a Bartington MS3 meter fitted with a MS2E surface scanning sensor, operated using Bartsoft software. The other half of the split-core was sliced into contiguous 1 cm (approximately 11.88 cm^3^) samples before being subsampled for loss-on-ignition (following [40], and a modified version of [41]), particle size analysis (pre-treated with H_2_O_2_, 35% w:v and (NaPO_3_)_6_, 5% w:v, grain size distribution measured using laser diffraction spectrometry performed by a Malvern Mastersizer 2000 equipped with a Hydro G dispersion unit, statistical analysis performed in Gradistat v8 [42] using the Folk and Ward (μm) summary output), charcoal and plant microfossil analysis (see below for details).

In the first instance, ‘bookend’ and exploratory radiocarbon dates were obtained for the longest cores recovered across the three basins. These initial results guided the selection of two cores (PTC3 and KKPC2) for detailed analysis, which were subsequently dated further. Overall, 28 samples were submitted for dating using AMS radiocarbon analysis (for details see Table 1). Macrophyte and wood fragments were hand-picked under low magnification and bulk sediment samples were extracted from the centre of the core with a scalpel. Those samples extracted from very organic or peat layers were pre-treated with KOH (10% w:v) to remove mobile humic acids [43], and all samples were oven-dried to a stable mass at 60°C prior to being submitted for AMS ^14^C analysis. Laboratory pre-treatment included acid-alkali-acid (AAA) washing. The obtained conventional radiocarbon ages were calibrated to calendar years using the SHCal13 curve [44] with an offset of -21 ± 6 years [45], to account for the mixing of Northern and Southern Hemisphere air-masses that occurs over monsoonal Southeast Asia [45–47]. This offset has been similarly applied in radiocarbon analyses elsewhere in southern and central Cambodia [25, 48, 49]. Chronological models for both sediment cores were built using the Bacon statistical package [50], using R [51] as an interface. Prior constraints, such as deposition rate and student-t distribution values were defined separately before running the model and were based on the stratigraphy and expected rates of accumulation.

**Table 1:** Details of each sample submitted for radiocarbon analysis. Includes conventional and calibrated ages (in years before present, using the SHCal13 calibration curve with an offset of -21 ± 6 years) plus errors. Modelled weighted mean (in calendar years C.E.) calculated using a Bacon age-depth model.

Core	Sample ID	Depth (cm)	Material dated	Radiocarbon age (^14^C yr BP ± 1σ)	Modelled calibrated age range CE (2σ or 95% probability)	Modelled weighted mean (CE)
**KKQC1**	D-AMS 006493	4–7	Wood fragment	572 ± 23	1324–1344 [12.5%]	n/a
					1388–1429 [82.4%]	
**KKQC2**	D-AMS 007104	43.5–47.5	Plant fragment	155 ± 30	1670–1749 [34%]	n/a
					1751–1783 [8.1%]	
					1795–1818 [9.6%]	
					1826–1894 [27%]	
					1907–1953 [16.1%]	
	D-AMS 007105	62.5–63.5	Organic mud	1010 ± 28	995–1009 [4.5%]	n/a
					1011–1053 [37.7%]	
					1059–1068 [2.8%]	
					1078–1147 [49.8%]	
	D-AMS 006494	103	Plant fragment	963 ± 24	1030–1155 [95%]	n/a
	D-AMS 007107	129–130	Organic mud	1224 ± 28	694–696 [0.2%]	n/a
					727–730 [0.3%]	
					766–896 [91.7%]	
					933–956 [2.8%]	
	D-AMS 007108	185–186	Organic mud	1143 ± 26	887–987 [95%]	n/a
	D-AMS 007106	275–276	Organic mud	1463 ± 32	550–557 [1.6%]	n/a
					571–655 [93.4%]	
	D-AMS 006495	281–282	Organic mud with plant fragments	1220 ± 20	772–886 [95%]	n/a
	D-AMS 006496	282–283	Organic mud with plant fragments	1026 ± 20	992–1047 [74%]	n/a
					1086–1133 [20.8%]	
**KKPC1**	D-AMS 006497	11–15	Woody twig	Modern	n/a	n/a
	D-AMS 006498	63–64	Peat	741 ± 26	1230–1249 [6.7%]	n/a
					1261–1312 [77.8%]	
					1359–1379 [10.4%]	
**KKPC2**	D-AMS 007109	10–11	Organic mud	128 ± 24	1685–1729 [21.8%]	1919
					1803–1952 [73.1%]	
	D-AMS 018422	19–20	Sediment (humates)	17 ± 22	1818–1827 [4.2%]	1871
					1897–1904 [2.1%]	
					1951–1955 [88.5%]	
	D-AMS 008223	28–29	Organic mud	Modern	n/a	n/a
	D-AMS 008224	46–47	Peat	55 ± 28	1700–1722 [7.8%]	1713
					1809–1838 [28.1%]	
					1844–1867 [6.9%]	
					1878–1933 [49.5%]	
					1937–1946 [2.2%]	
					1954–1953 [8.7%]	
	D-AMS 018423	51–52	Twig fragments	170 ± 24	1667–1712 [23.9%]	1679
					1718–1813 [47%]	
					1836–1849 [4.4%]	
					1851–1890 [10.9%]	
					1924–1953 [8.7%]	
	D-AMS 007110	60–61	Peat	528 ± 28	1400–1445 [95%]	1436
**PTC3**	D-AMS 006499	10–18	Leaf	Modern	n/a	n/a
	D-AMS 006500	95–96	Peat	345 ± 27	1481–1636 [95%]	1436
	D-AMS 006501	111–112	Peat	566 ± 22	1327–1340 [5.5%]	1607
					1390–1432 [89.4%]	
	D-AMS 007111	125–126	Peat	400 ± 25	1448–1511 [66.4%]	1509
					1550–1557 [1.2%]	
					1574–1622 [27.3%]	
	D-AMS 007112	173–174	Organic mud	758 ± 29	1223–1301 [93.1%]	1239
					1367–1373 [1.8%]	
	D-AMS 006502	181–184	Leaf fragment	Modern	n/a	n/a
	D-AMS 006503	213–214	Organic mud	1234 ± 26	692–700 [1%]	841
					718–731 [1.5%]	
					765–890 [92.4%]	
**PTC4**	D-AMS 007113	21–22	Peat	340 ± 27	1485–1638 [95%]	n/a
	D-AMS 007114	64–65	Organic mud	1026 ± 21	992–1047 [72.1%]	n/a
					1085–1135 [22.8%]	
	D-AMS 007115	118–119	Organic mud	1201 ± 23	772–898 [82.1%]	n/a
					930–961 [12.8%]	
	D-AMS 007116	121–122	Organic mud	971 ± 22	1031–1150 [95%]	n/a

Particulate charcoal–the product of the incomplete combustion of carbonaceous material–remains preserved in sedimentary archives over long (geological-scale) time periods [52, 53], and therefore presents as a robust proxy for the history of burning in a landscape. However, interpreting charcoal records is inherently complex, as these archives capture both natural [54, 55] and anthropogenic [25, 56] fire events, which occur over various spatial scales, and are modulated by complicated transport and dispersal processes [57, 58].

Allocating fire events to their corresponding source areas relies on the relationship between the size of the particle interred and the distance from the source fire. As such, charcoal records can be separated into size classes, with each class representing different, but overlapping catchment scales. Following their release from the source fire and convection into the air column, larger particles (i.e. > 100 μm) with higher settling velocities generally fall out first and are thus deposited within close (30 m to 10 km) range of the burning event, while smaller particles tend to be transported over longer distances [59]. As such, in this study microscopic (> 106 μm) and two macroscopic (106–250 μm and > 250 μm) charcoal fractions, corresponding roughly to extra-regional, regional-local and local sources areas respectively, were isolated for analysis. Extraction and analysis protocols followed a modified version of Stevenson and Haberle [60]. 2 cm^3^ sediment samples (at 2–3 cm resolution) were disaggregated using (NaPO_3_)_6_ (5% w:v), mixed mechanically for a minimum of four hours, and then wet sieved through 250 μm and 106 μm steel mesh.

For the macrocharcoal size fractions, the entirety of each sample was then transferred to a glass petri dish, placed over 1 cm grid graph paper and counted (systematically, one grid square at a time) under 40 x magnification using a binocular microscope. Absolute values (particles cm^-3^) were converted into influx values (particles cm^-2^ year^-1^) using the method specified in Mooney and Tinner [61]. The microcharcoal fraction was distilled by digesting carbonates (HCl 10% w:v), silicates (HF 45% w:v), and all extraneous organic matter–leaving behind the chemically-inert charred particles and sporopollenin (acid hydrolysis mixture of a 9:1 ratio of (CH_3_CO)_2_O and H_2_SO_4_). As a final step, samples were mixed with a glycerol medium and mounted on glass microscope slides, before being counted under 400 x magnification. Lycopodium spores were added to each sample during chemical treatment, and the number of microcharcoal fragments were counted along a transect until 25 marker spores had been tallied. Microcharcoal concentrations (grains cm^-3^) were calculated using the standard approach outlined in Maher [62], which were subsequently converted to influx values (particles cm^-2^ year^-1^) (see [61]) for analysis.

Reconstructions of past fire regimes also require the distinction of ‘peak’ fire signals from long-term levels or background ‘noise’ [63–66]. Peak fires are considered to represent individual fire events or periods occurring proximal to the site, while the background regime is largely the result of the combined, long-term, distal fire history operating in the regional landscape. This separation is achieved on this non-contiguous dataset through a basic statistical analysis, namely by calculating the deviation of charcoal influx from the long-term (i.e. length of record) mean. Peak fire periods were then inferred from positive influx values.

Applying a multi-proxy palaeoecological approach, in particular utilising plant microfossil analyses, can also disentangle the human-induced signal from the background, natural fire regime, especially when applied in conjunction with known archaeological and long-term climate records for the region. As such, charcoal analysis also involved comparison against three climate records from the Vietnamese highlands [67], tropical India [68], and southern China [69], and will be placed within the context of the archaeological history of the city.

Plant microfossil (pollen and spore) concentration followed the standard treatment protocol (see [70]; see also description of microcharcoal treatment above). Pollen and spores were classified and counted under 400 x magnification using standard transmitted light microscopy at depths of 10 cm down the core. Resolution was increased to 3–5 cm in segments of the core where distinct change in the pollen assemblage was evident. Specimen classification was aided by a reference compilation of known Cambodian plant species [71] collated from the palynomorph photographs and descriptions of Maxwell [72], the Australasian Pollen and Spore Atlas [73], Huang [74], Roubik and Patino [75], Penny [76], Hamilton [77] and a collection of pollen from dry deciduous and semi-evergreen forests of north-east Thailand housed at the Rijksherbariam/Hortus Botanicus in Leiden, the Netherlands. Pollen taxa are expressed as the relative percentage of either arboreal (trees and woody shrubs) or herbaceous (both dryland and wetland) taxon groups and are plotted stratigraphically using C2 software v1.7.6 [78]. Stratigraphically constrained cluster analysis was performed to differentiate zones of significant transition in the data, using the R packages ‘rioja’ v0.9.6 [79] and ‘vegan’ v2.2.1 [80]. These statistical packages employ the incremental sum-of-squares (CONISS; [81]) and Euclidean distance methods to ‘cluster’ the pollen data and a broken stick model [82] to determine the number of significant zones.

To aid in the interpretation of landscape change from the pollen data, a Principal Component Analysis (PCA) was also performed to decompose the multivariate dataset. Data were initially normalised by dividing absolute values by their standard deviation. PCA analysis was undertaken using the ‘stats’ package v3.4.1 in R.

## Results

### Sedimentology and chronology

**Fig 3:**
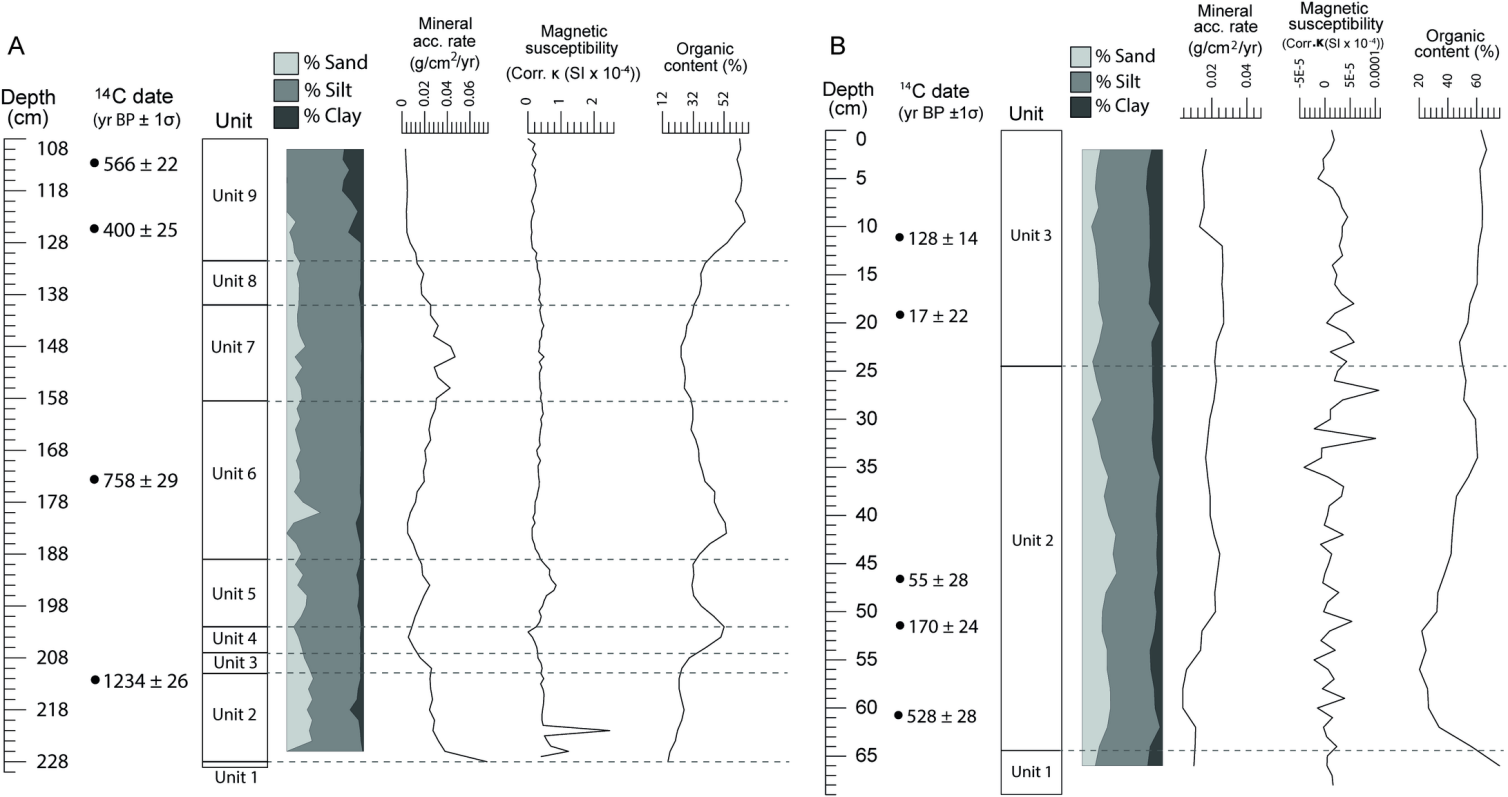
Core stratigraphic and sedimentary data. Data includes proportions of sand/silt/clay, mineral accumulation rates, magnetic susceptibility readings, and organic content (loss-on-ignition). **A**: Core PTC3, **B**: Core KKPC2.

Core PTC3 is 229 cm in length comprising ten stratigraphic units (see Fig 3A). The basal Unit 1 (229–228 cm depth) is a very dark greyish brown [2.5Y/3/2] wet, massive, sapropelic mud with a sharp, wavy upper boundary. Unit 2 (228–211 cm depth) is a very dark greyish brown [2.5Y/3/2] wet, massive, sapropelic, slightly gravelly sandy mud to sandy mud with indistinct, planar upper boundary. Unit 3 (211–207 cm depth) is a very dark grey [2.5Y/3/1] wet, massive, sapropelic, sandy mud with a gradational, planar upper boundary. Unit 4 (207–202 cm depth) is an olive black [2.5Y/2.5/1] wet, massive, sapropelic sandy mud with a gradational, planar upper boundary. Unit 5 (202–189 cm depth) is a very dark greyish brown [2.5Y/3/2] wet, massive, sapropelic, slightly gravelly sandy mud to sandy mud with an indistinct, planar upper boundary. Unit 6 (189–158.5 cm depth) is a very dark grey [2.5Y/3/1] wet, massive, sapropelic, sandy mud containing a few large organic fragments and with an indistinct, planar upper boundary. Unit 7 (158.5–140 cm depth) is a very dark greyish brown [2.5Y/3/2] wet, massive, sapropelic, sandy mud with an indistinct, planar upper boundary. Unit 8 (140–131.5 cm depth) is a very dark grey [2.5Y/3/1] wet, massive sapropelic sandy mud with a gradational, smooth upper boundary. Unit 9 (131.5–108 cm depth) is a black [2.5Y/2.5/1] wet, massive, sapropelic mud with a sharp, planar upper boundary. This is overlain by Unit 10 (108–0 cm depth), which is a black [2.5Y/2.5/1], wet, massive, herbaceous peat containing many large organic fragments. This uppermost 108 cm is highly unconsolidated and had mixed either during extraction or after the core had been cut into separate lengths for transport, and was thus discarded from any further analysis.

Core PTC3 is dominated by silt-sized particles (on average, 76.8 ± 9.1% silt). In general, the proportion of sand deposited into the moat decreases upwards through the core, with sand being almost absent in the top 14 cm. Mineral accumulation rates are weakly positively correlated (r = 0.447) with magnetic susceptibility and strongly negatively correlated with organic content (r = -0.847), showing that there appears to be definite shifts between episodes of allochthonous siliciclastic material deposition and autochthonous organic matter input into the moat. Magnetic susceptibility shows the greatest variation between 228–188 cm (σ = 0.374 SI x 10^−4^ compared to 0.111 SI x 10^−4^ for the remainder of the core) and two strong peaks occur toward the base of the core, presumably representing brief episodes of substantial mineral influx, as these peaks occur in Unit 2 where mineral accumulation rates are high. Highest and least variable organic content input (mean = 62.3 ± 1.9%) occurs in the top 18 cm of the core.

Core KKPC2 is 69 cm in length comprising three stratigraphic units (see Fig 3B). The basal Unit 1 (69–64.4 cm depth) is a very dark grey [2.5Y/3/1], moist, massive, sapropelic, sandy mud with an abrupt, wavy upper boundary. Unit 2 (64.4–24.5 cm depth) is a black [2.5Y/2.5/1], moist, massive peaty, sandy mud containing many large and very large woody and other organic fragments, and with a clear, smooth upper boundary. Organic/woody fragments are particularly concentrated between 41 and 44 cm depth. This is overlain by Unit 3 (24.5–0 cm depth), which is a very dark grey [2.5Y/3/1], moist, massive, peaty, sandy mud with black, distinct, irregular to platy mottles throughout.

Core KKPC2 is highly organic, particularly so in Units 1 (75.9%) and Units 3 (63.8 ± 1.6% between 0 and 8 cm depth). There is a positive relationship between organic content and mineral accumulation rate in this record (r = 0.319) as gross accumulation rates increase upwards through the core. Magnetic susceptibility is low and variable throughout the core (mean = 0.17 ± 0.24 SI x 10^−4^) and shows no clear relationship with any other variable. In general, relative proportions of sand (mean = 24.8 ± 8%), silt (mean = 63.1 ± 6.1%) and clay (mean = 12.1 ± 4.6%) remain fairly consistent over depth, with the proportion of sand decreasing slightly upwards through the core.

Radiocarbon results are presented in Table 1. KKPC2 and PTC3 were selected for further analysis based on these results, as together they presented the most extensive time coverage and relatively few temporal inversions during the initial exploratory dating. The basal calibrated date from PTC3 (D-AMS 006503), based on bulk carbon taken from a sapropelic sandy mud layer (Unit 9), returned an age of 692–890 C.E. (weighted mean of 841 C.E.). As this sample came from a depth of 214 cm, which is 15 cm from the base of the core, the age-depth model suggests that organic sedimentation within the moat of Prasat Thom began prior to the 9th century CE, and possibly as early as the late 7th century CE. As we discarded the top 108 cm of PTC3 due to its mixing (see sedimentology description above), only samples D-AMS 006501, D-AMS 007111, D-AMS 007112 and D-AMS 006503 were included in the Bacon age-depth model. Bacon identified a single date, D-AMS 006501, as an outlier, which was thus ‘bypassed’ in the final model.

**Fig 4:**
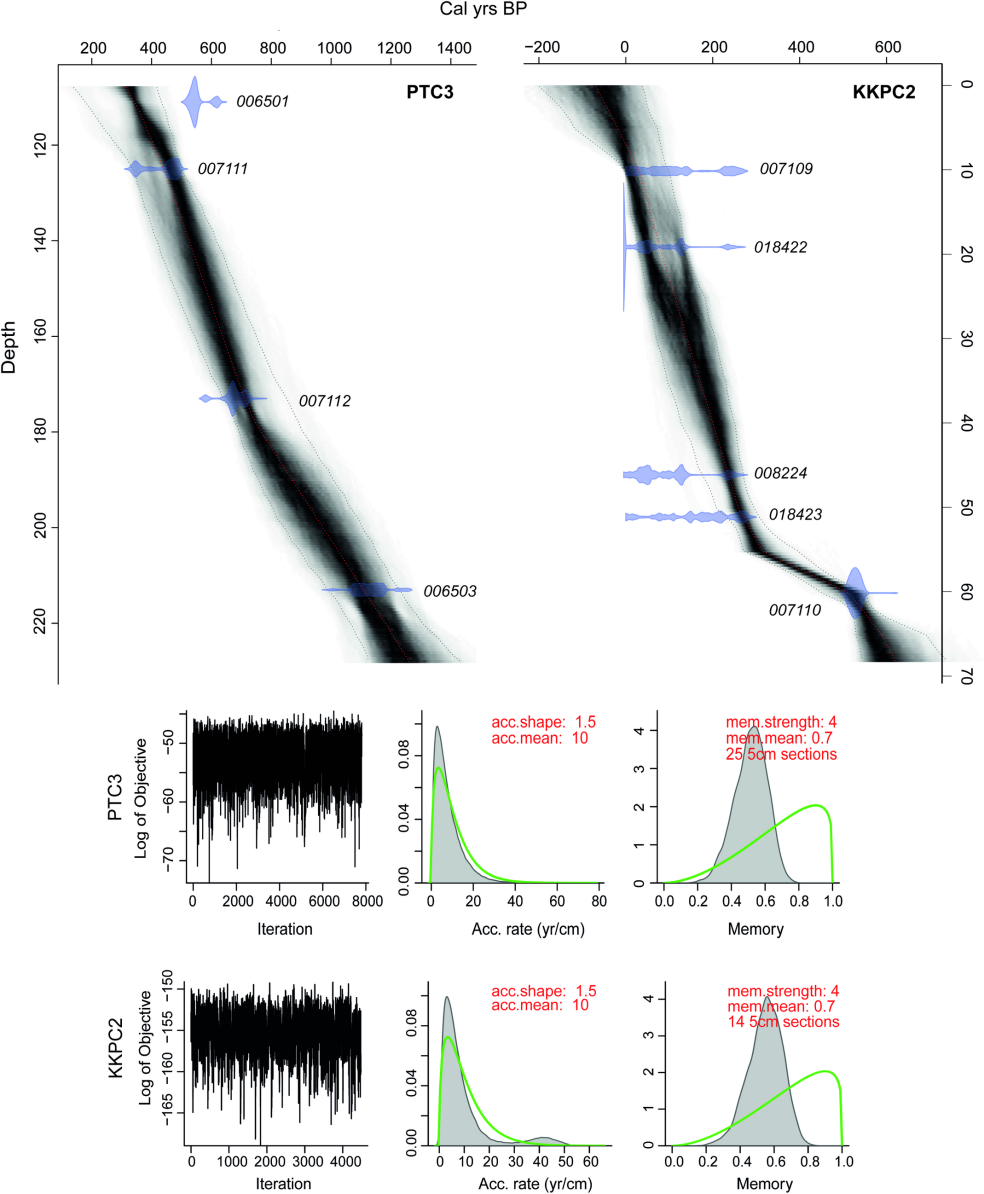
Bacon age-depth models for both cores analysed. **Top**: Calibrated radiocarbon ages are included individually in pale purple. Red curve indicates the weighted mean age for each modelled depth and grey stippled lines indicate the 95% confidence intervals for the final model. **Bottom**: left panels depict the MCMC iterations. Prior and posterior information is included in the centre and right panels, including accumulation rate (centre) and the variability in the accumulation history (right).

Fig 4 shows the Bacon model for KKPC2. By default, the model applies a student’s t distribution to accommodate ages with very wide range distributions, however to restrict the model through the basal date (D-AMS 007110) for KKPC2 it was necessary to alter the student-t values (*t*.*a* and *t*.*b*) here, from 3 and 4 to 33 and 34 respectively (see [83]). Considering the overlapping calibrated age ranges of D-AMS 007109, D-AMS 018422, D-AMS 008224 and D-AMS 018423, it is probable that deposition of these sediments occurred within a very short period, and thus represents a shift to rapid and recent bulk accumulation within the reservoir.

### Plant microfossil and charcoal

**Fig 5:**
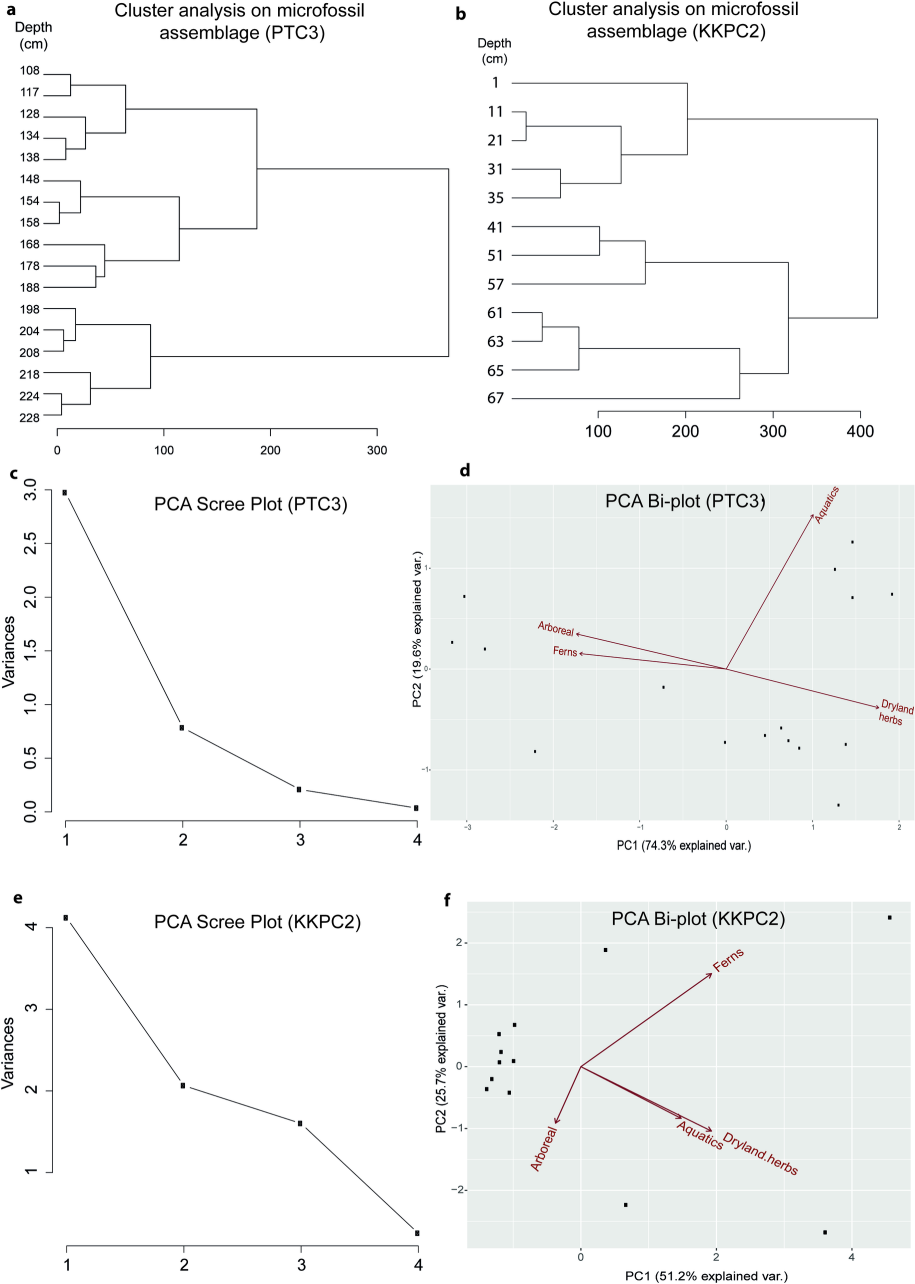
Statistical analysis on microfossil assemblages for both cores. **a**/**b**: stratigraphically constrained cluster analysis (CONISS) to determine the number of zones analysed in the pollen data; **c**/**d**: results of Principal component analysis, including scree plot and bi-plots for core PTC3 and **e**/**f**: core KKPC2.

**Fig 6:**
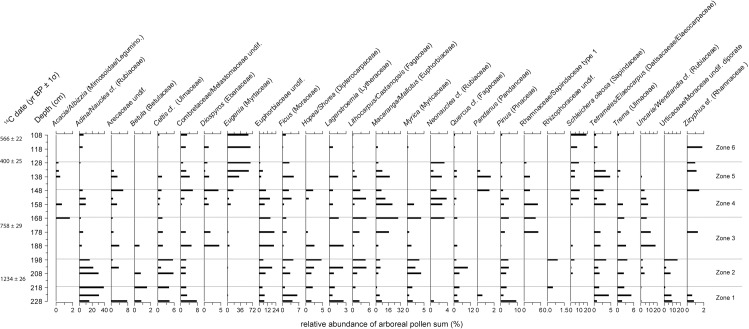
Relative abundances of pollen taxa observed in core PTC3. Includes only trees and woody shrubs. ‘cf.’ and ‘sf’ refer to our confidence in a species’ identification, and stand for ‘comparable form’ and similar form’ respectively.

**Fig 7:**
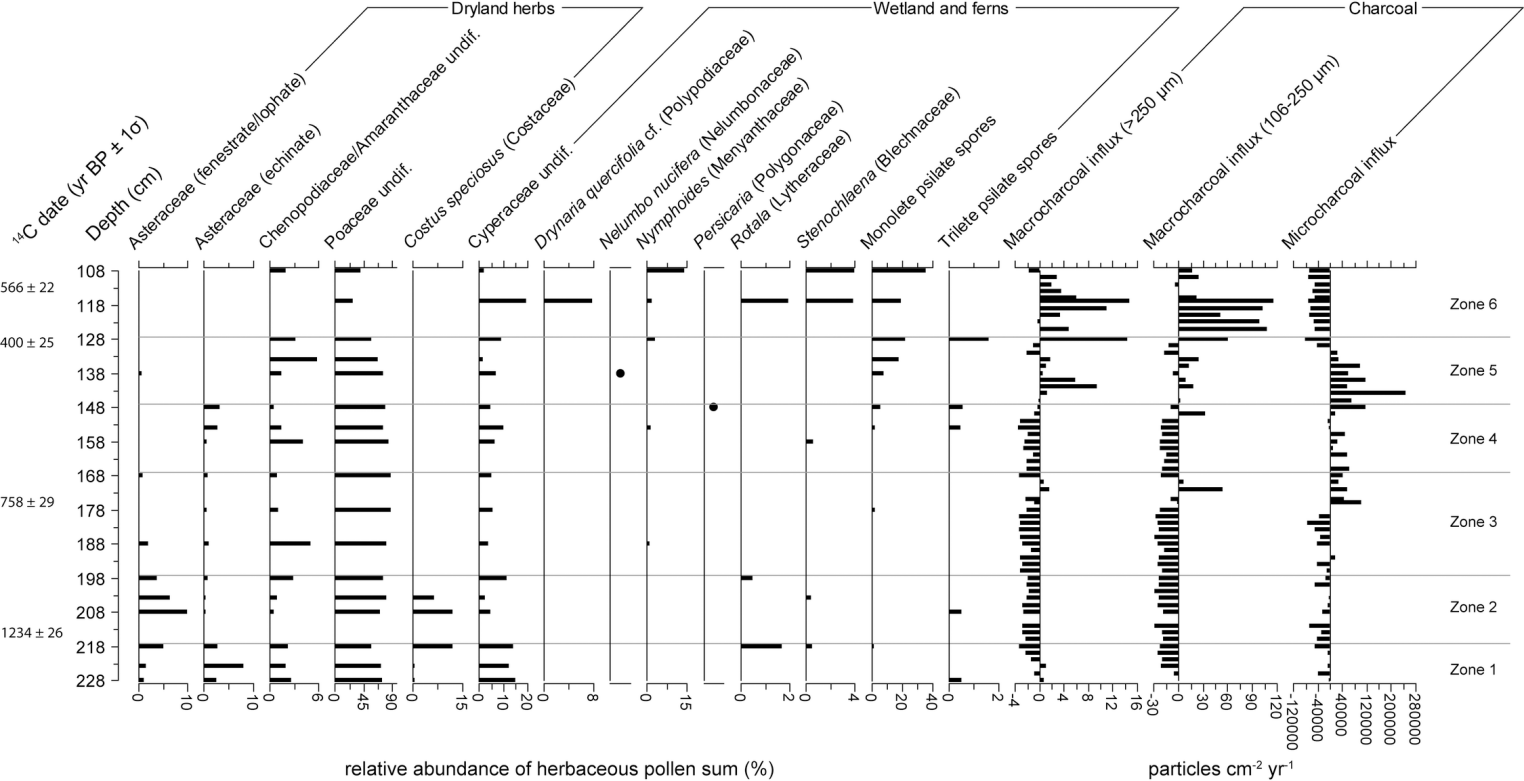
Relative abundances of dryland and wetland herbaceous pollen and fern spore taxa observed in core PTC3. Also includes charcoal influx data of all three size classes analysed, presented as residuals from the long-term mean. ‘cf.’ and ‘sf’ refer to our confidence in a species’ identification, and stand for ‘comparable form’ and similar form’ respectively.

Eighty pollen and spore types were identified from 17 samples between 108 and 228 cm of core PTC3. Total plant microfossil counts per depth ranged between 299 and 361, with an average of 322. Microfossil influxes were, in general, very low. Classification of the pollen data (see Fig 5A) identified six zones of differing vegetation assemblage (Figs 6 and 7). *Adina/Nauclea* cf. dominates the arboreal assemblage in Zones 1 and 2 (229–198 cm or 689–998 CE). Urticaceae/Moraceae, *Ficus*, *Trema*, *Myrica* and Euphorbiaceae, as well as extra-regional forest taxa such as *Celtis* cf., *Pinus* and *Quercus* cf. are also common. Dryland herbs, in particular Asteraceae and *Costus speciosus*, are at their most abundant in these first two zones. While Cyperaceae is relatively abundant, other wetland taxa and ferns are infrequent. In Zone 3 (198–168 cm or 998–1268 CE), many of the taxa dominant in both the arboreal and herbaceous assemblages of the previous two zones diminish and are replaced by *Diospyros*, *Macaranga/Mallotus*, *Uncaria/Wendlandia* cf., Rhamnaceae/Sapindaceae type 1, Poaceae and Chenopodiaceae/Amarantheaceae. Euphorbiaceae, however, remains a prominent forest component, and herbaceous swamp taxa remain very low. In Zone 4 (168–148 cm or 1268–1380 CE), a distinct change occurs, as *Schleichera oleosa* and *Eugenia* begin to increase in relative abundance. These two species will go on to dominate the arboreal pollen record in Zones 5 and 6 (above 148 cm, after 1380 CE). *Neonauclea* cf., *Pandanus*, Combretaceae/Melastomaceae and *Tetrameles/Elaeocarpus* also increase in Zones 4 and 5. A similar shift occurs in the herbaceous assemblage, where the dominance of Poaceae gradually reduces through Zone 5, as the abundance of ferns and other aquatic taxa (*Nymphoides*, in particular) increase through the remainder of the record.

Charcoal influx (Fig 7), across all three size classes, remain below the long-term mean throughout Zones 1, 2 and the majority of Zone 3. The influx of microcharcoal remains above average throughout Zones 4 and 5, and peaks at 136 cm (1447 CE), before declining again in Zone 6. Macrocharcoal influx is highly variable (>250 μm influx mean = 3.846 ± 4.135, 106–250 μm influx mean = 32.324 ± 35.721 particles cm^-2^ yr^-1^). While isolated peaks in macrocharcoal occur in Zone 3 (at 170–172 cm, 1257–1245 CE) and Zone 4 (150 cm, 1368 CE), sustained increases in both size fractions do not occur until Zones 5 and 6. The burning signal throughout Zone 6 is particularly strong.

**Fig 8:**
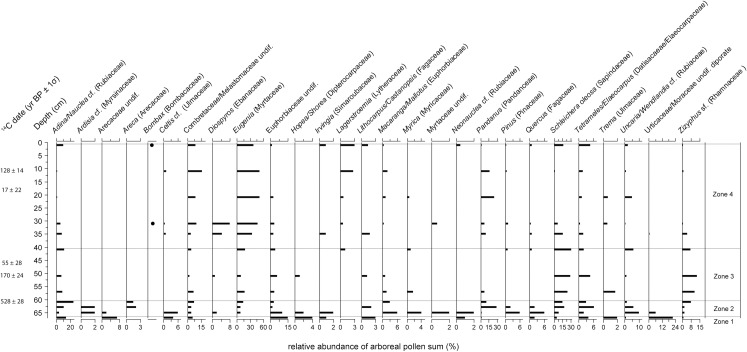
Relative abundances of pollen taxa observed in core KKPC2. Includes only trees and woody shrubs. ‘cf.’ and ‘sf’ refer to our confidence in a species’ identification, and stand for ‘comparable form’ and ‘similar form’ respectively.

**Fig 9:**
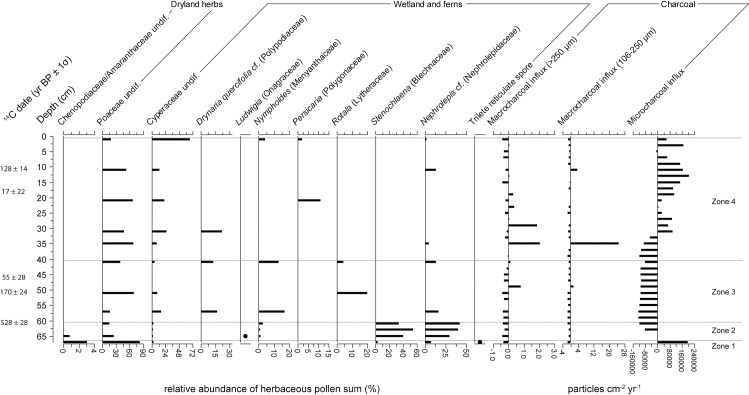
Relative abundances of dryland and wetland herbaceous pollen and fern spore taxa observed in core KKPC2. Also includes charcoal influx data of all three size classes analysed, presented as residuals from the long-term mean. ‘cf.’ and ‘sf’ refer to our confidence in a species’ identification, and stand for ‘comparable form’ and ‘similar form’ respectively.

For core KKPC2, sixty-five pollen and spore types were identified from 12 samples between 1 and 67 cm. Total plant microfossils counted per depth ranged from 306 to 687, with an average of 417. Cluster analysis (see Fig 5B) identified four vegetation zones (Figs 8 and 9). Zone 1 includes only one sample, at 67 cm (1340 CE), and largely comprises *Adina/Nauclea* cf., Arecaceae, Combretaceae/ Melastomaceae, Euphorbiaceae, *Hopea/Shorea*, *Lithocarpus/Castanopsis*, *Tetrameles/Elaeocarpus*, *Trema* and Urticaceae/Moraceae. Poaceae and Chenopodiaceae/Amarantheaceae dominate the herbaceous taxa. The most diverse range of taxa exists through Zone 2 (67–60 cm or 1340–1437 CE), where, in addition to the taxa represented in Zone 1, *Areca*, *Macaranga/Mallotus*, *Myrica*, *Pandanus*, *Schleichera oleosa* also comprise the forest assemblage. Of the herbaceous taxa, the ferns *Stenochlaena* and *Nephrolepis* cf. clearly dominate. *Eugenia* also appears in the record for the first time, but in relatively low numbers. A sharp transition occurs between Zones 2 and 3, where many forest species dominant in Zone 1 and 2 decline dramatically. Zone 3 (60–40 cm or 1437–1749 CE) sees a reduction in ferns, beside *Drynaria quercifolia* cf., which increases along with the aquatics *Nymphoides* and *Rotala*. *Schleichera oleosa* and *Zizyphus* sf. increase as components of the arboreal sum. Zone 4 (above 40 cm, after 1749 CE) sees an increase in Cyperaceae and isolated and non-concurrent peaks in other wetland taxa (*Drynaria quercifolia* cf., *Persicaria*, *Nymphoides*, and *Nephrolepis* cf.) and marked increases in *Eugenia*, Combretaceae/Melastomaceae, and *Pandanus*.

A clear trend in the microcharcoal data is evident in KKPC2 (Fig 9). Besides a peak in Zone 1 at 67 cm (1340 CE), below average influx levels are sustained throughout Zones 2 and 3, before a clear shift to consistently above-average influxes occur within Zone 4 (at 31 cm or 1802 CE), which are maintained for the rest of the record. The macrocharcoal record shows no distinct trend, however. Overall macrocharcoal levels were very low and highly variable (>250 μm influx mean = 0.382 ± 0.559, 106–250 μm influx mean = 1.595 ± 4.486 particles cm^-2^ yr^-1^). Isolated peaks occur at 49 cm (1696 CE), 35 cm (1779 CE) and 29 cm (1814 CE).

### Statistical analysis

The PCA results for PTC3 identified that the first two components explain almost 94% of the variance in the summary pollen data, and a scree plot confirms that these two components are the most significant (eigenvalues greater than 0.78) (Fig 5C). Variable loading scores indicate that dryland herbs (0.55) and arboreal (trees and woody shrubs) (0.54) contribute strongly to the distribution of values along the PC1 axis (Fig 5D), and this component can be taken to represent the degree of land clearance in the pollen catchment. Aquatic flora (containing a loading score of 0.94) contributes most strongly toward the distribution of values along the PC2 axis. The growth of aquatic vegetation reflects the development of localised swamp vegetation within the moat itself, and thus was used as an indicator of the abandonment of intensive water management practices within the temple city.

The PCA results for KKPC2 identified that the first three components account for almost 97% of the variance in the summary pollen data. A scree plot reveals that, considering components with eigenvalues higher than 1, these first three components are the most significant (Fig 5E). Variable loading scores indicate that the strongest contribution to the distribution of sample values along the PC1 axis is almost equally shared by dryland herbs (0.62) and ferns (0.62), while ferns (0.68) most strongly influence PC2 values and aquatics (0.68) and dryland herbs (0.62) together have the most influence on the axis of PC3 (Fig 5F). As such, PC1 will be taken to represent change in land use and occupation, PC2 as reservoir abandonment, and PC3 as a general metric for the attenuation of land use or occupation.

## Discussion and conclusion

**Fig 10:**
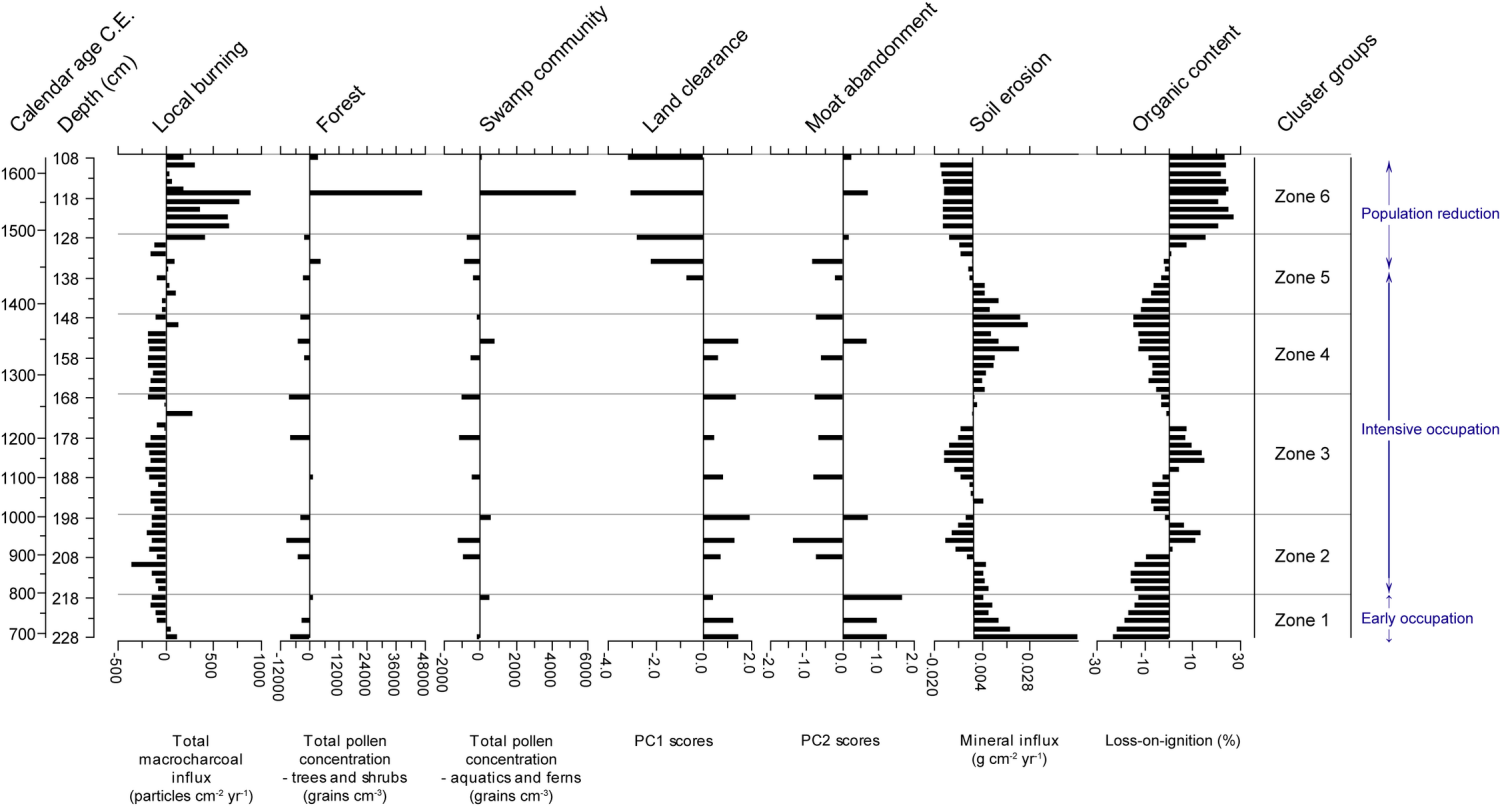
Summary palaeoecological data for Prasat Thom. Land use and human activity indices (land clearance and moat abandonment) calculated using Principal Component Analysis. Local burning index calculated through the addition of the two macrocharcoal size fractions (>250 μm and 106–250 μm).

Figs 10 (Prasat Thom) and 11 (Andong Preng) present a summary of the variables used to interpret landscape change around each site. Over the entire record, both macro- and microcharcoal results for both sites are not well-correlated (see Table 2 and Fig 12) with extra-regional climate data. As such, charcoal records presented here can be considered a product of either stochastic processes or human activity rather than direct climate forcing. It is possible that changing types of available vegetation has influenced the burning regime, however the correlation between the charcoal and woody vegetation pollen records for Andong Preng is very low (r = 0.08). The correlation is relatively high for Prasat Thom (r = 0.73), however if the overwhelming (60%) influence of *Eugenia* pollen on the arboreal pollen sum is removed from the analysis, the correlation reduces substantially (r = -0.38). *Eugenia* species grow along the margins of these reservoirs, and are known components of swamp forests in Cambodia [84], and as such most likely represent a local pollen signal in this record rather than a strong component of the regional forest fuel load. In this case, it is probable that low intensity and muted variability in burning activity represents active and intensified management of the landscape, in particular a change from shifting cultivation strategies and wide-spread forest burning to rice-growing within permanent, bunded fields and lower-intensity, controlled burning of herbaceous vegetation associated with agriculture (see also [85, 86]). The shift toward greater and more variable fire activity in the macrocharcoal records presented here, therefore, likely signals severe reductions in these land management practices.

**Fig 11:**
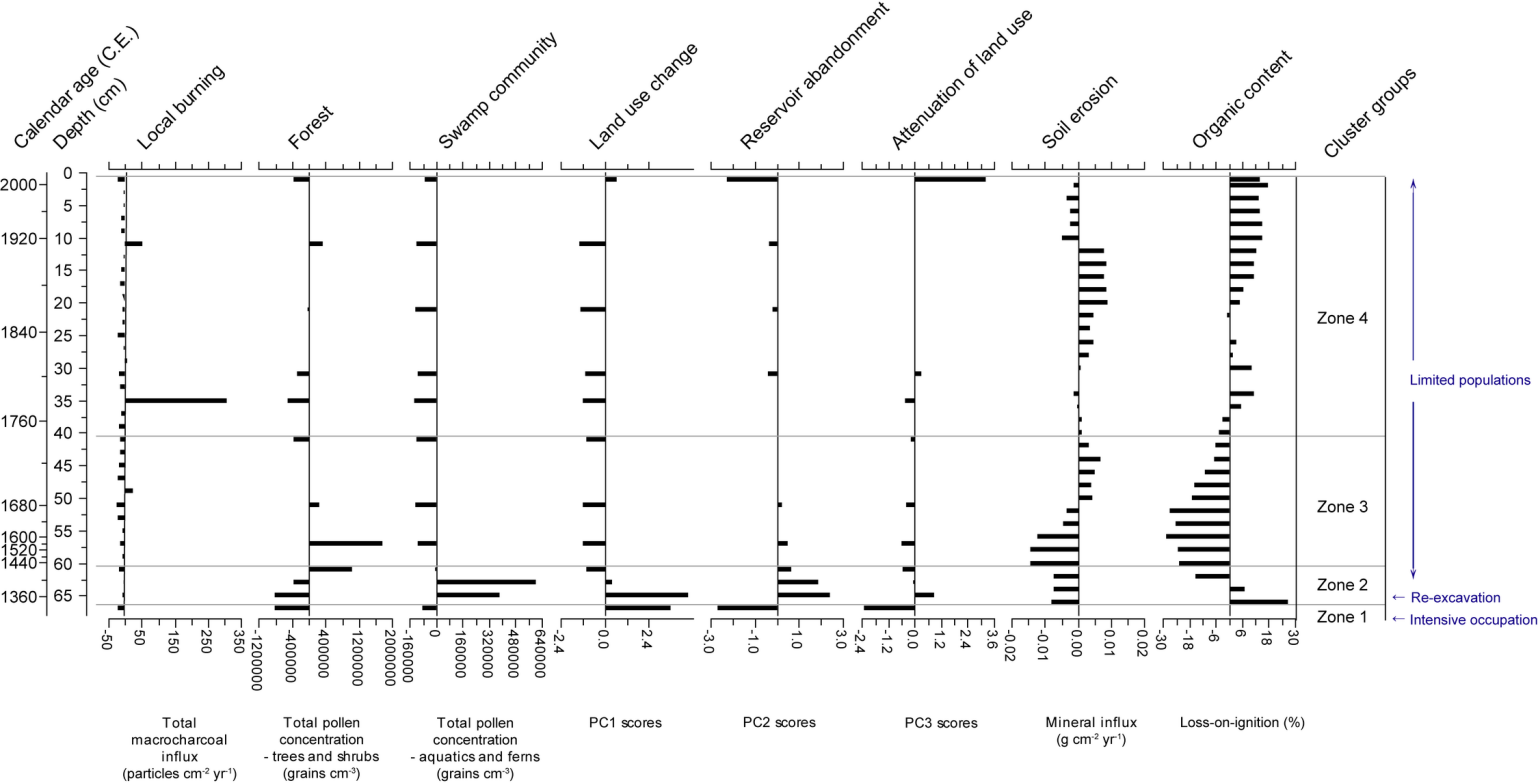
Summary palaeoecological data for Andong Preng. Land use and human activity indices (land use change, reservoir abandonment and attenuation of land use) calculated using Principal Component Analysis. Local burning index calculated through the addition of the two macrocharcoal size fractions (>250 μm and 106–250 μm).

**Fig 12:**
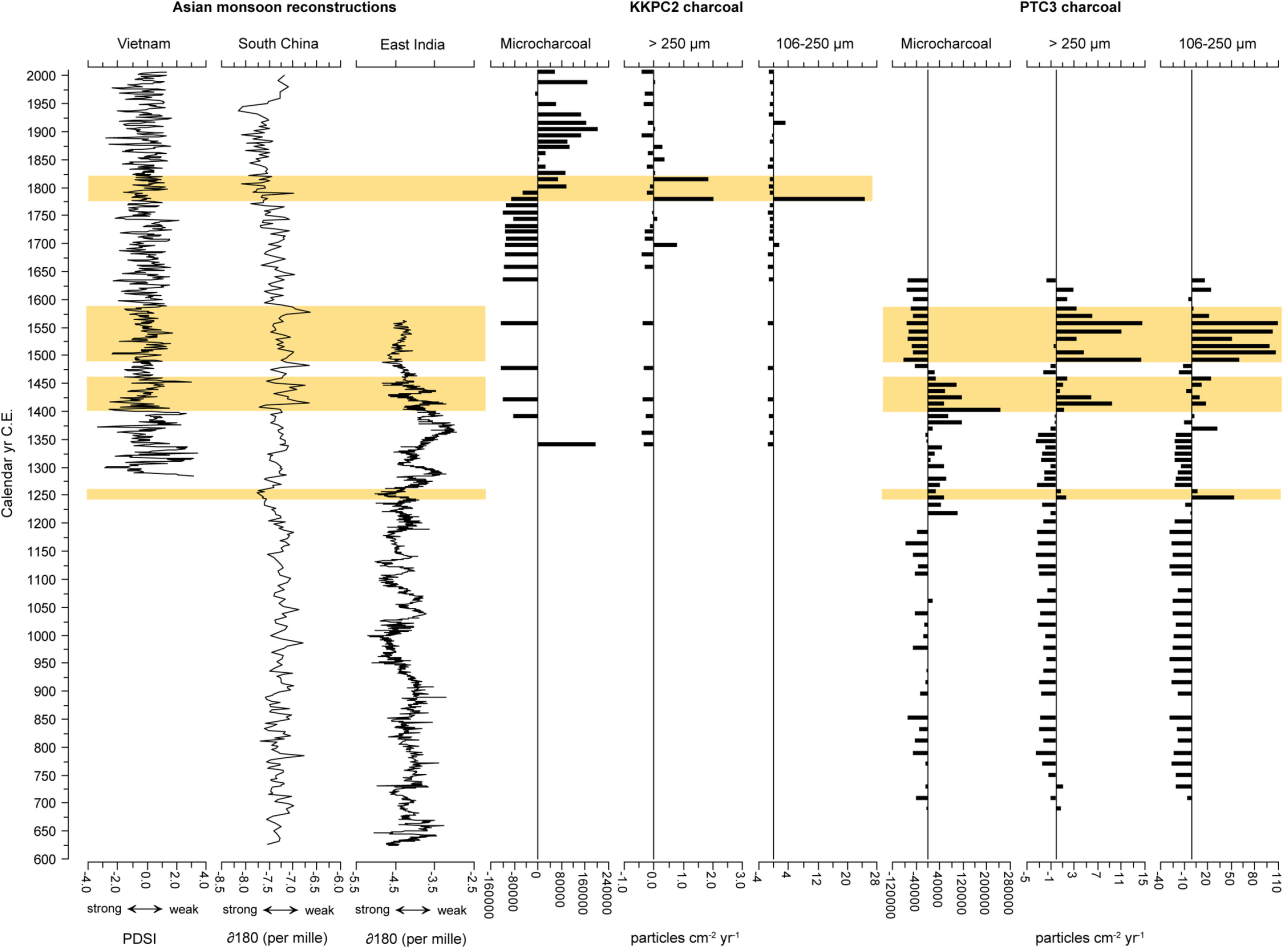
Published hydro-climate records representing extra-regional climate plotted against macro- and micro-charcoal records for each core analysed. Charcoal influx is presented as the deviation from the long-term (whole-of-record) mean. Hydro-climate records (representing the relative strength of Asian monsoon systems) are from (left to right) [67], [69] and [68]. Yellow bars represent distinct events or periods of heightened fire activity.

**Table 2:** Correlation analysis between charcoal records and regional hydro-climate records. Southern China data supplied by [69], Highland Vietnam by [67], and Tropical India by [68].

Core (*site*)	Size fraction	Hydroclimate record	Correlation coefficient
**PTC3 (*Prasat Thom*)**	Microcharcoal	Southern China	0.008
		Highland Vietnam	-0.035
		Tropical India	0.380
	Macrocharcoal (>250 μm)	Southern China	0.093
		Highland Vietnam	-0.199
		Tropical India	-0.106
	Macrocharcoal (106–250 μm)	Southern China	0.017
		Highland Vietnam	-0.190
		Tropical India	-0.173
**KKPC2 (*Andong Preng*)**	Microcharcoal	Southern China	-0.854
		Highland Vietnam	0.083
		Tropical India	0.322
	Macrocharcoal (>250 μm)	Southern China	-0.471
		Highland Vietnam	-0.178
		Tropical India	-0.083
	Macrocharcoal (106–250 μm)	Southern China	0.315
		Highland Vietnam	-0.147
		Tropical India	0.757

### Prasat Thom

By combining results from both macrocharcoal fractions, Fig 11 shows that, from the 8th to late 14th centuries, local fire activity is consistently below the long-term average (mean residual = -123.45, σ = 102.72 particles cm^-2^ yr^-1^). Following this, a transition period occurs throughout the 15th century, where burning levels remain closer to the mean and slightly less variable (mean residual = -9.81, σ = 100.36)). From the late 15th century, and continuing throughout the 16th and early 17th centuries CE, local fire activity increases in both magnitude and variability (mean residual = 415.45, σ = 292.53), suggesting the attenuation of intensive agricultural land uses.

Coinciding with the transition period in the burning data in the early to mid-15th century is a marked shift from consistently high to consistently low levels of land clearance. Soil erosion is at its highest levels at the beginning of the record, presumably during and immediately following the excavation of the moat in the late-7th to early 8th century. Continued maintenance of the moat, however, does not appear to begin until the late 9th century (perhaps foreshadowing royal occupation of the city) but remains prevalent until the mid-15th century. Together these indices suggest that occupation of Koh Ker was relatively intensive between at least the 9th century and the mid to late 15th century.

Intriguingly, two periods of low soil erosion and high organic content, suggesting vegetation growth within the moat, occurs during the 10th (during the supposed height of the city’s occupation) and 12th centuries. However, these periods also coincide with low indices for moat abandonment and swamp community abundance, and high levels of land clearance, suggesting that land use intensities and water infrastructure maintenance remained high at these times.

The most fundamental shifts in all indicia began to occur by the mid-15th century at Prasat Thom. In addition to an increase in the frequency/intensity, as well as variability, of local burning, indicia for land clearance greatly reduce in the mid-15th century. Soil erosion seems to stabilise to well below average levels, and maintenance of the moat appears to have been abandoned as herbaceous swamp species proliferate and organic material derived from them accumulates unabated within the moat. In concert, this evidence suggests that, by the mid-15th century, agricultural and administrative populations within the city of Koh Ker had begun to decline and ongoing management of the city, in line with Angkor-period water management techniques, ceases, at least for the central temple of Prasat Thom.

### Andong Preng

The summary data for KKPC2 suggests that the reservoir had been constructed by at least the early 14th century (Fig 11). As the KKPC2 core failed to reach basal sediments, however, this date is unlikely to reflect Andong Preng’s initial excavation, which, if it was indeed associated with the Royal Palace, may have instead occurred during the early to mid-10th century. Continual management (e.g. clearance) of the reservoir during the city’s royal occupation may have eradicated the earliest section of this sediment record. Moreover, the fact that the Zone 2 contains the highest organic content for the record (up to 75.8%), as well as a peak in herbaceous swamp taxa, suggests that Zones 2 to 4 have captured the land use history of this city that post-dates a period of cessation of maintenance and active utilisation of this reservoir. At the boundary of Zones 1 and 2 a hiatus very possibly occurs, despite no clear evidence for an unconformity at this point in the stratigraphy. However, a drastic change in the pollen assemblage (see Figs 8 and 9), organic content and several land use change metrics (Fig 11) suggest that sedimentation was interrupted here through re-excavation and clearance of the reservoir. The sharp peaks in organic content and herbaceous swamp community taxa (also represented by the reservoir abandonment component), likely represents the interment of fragments of the floating vegetation mat that had been disturbed during the 14th century re-excavation.

Following re-excavation, several indicators suggest sustained reoccupation of the landscape, albeit on a greatly reduced scale. The abundance of swamp community growing within the reservoir declines and remains below the long-term mean for the remainder of the record. The proxy for reservoir abandonment similarly declines and maintains very low variability throughout Zone 3 and most of Zone 4. Soil erosion appears to increase gradually and remain above the long-term average throughout this period, and the land use change component undergoes a marked and sustained shift from positive to negative values. Local fire activity, for the most part, remains low and with minimal variability, again likely denoting the sustained maintenance of an agrarian landscape. High organic content throughout the 20th century coincides with low soil erosion levels (correlation between mineral influx and organic content r = -0.623), where it is presumed that the development of a floating vegetation mat has begun to winnow out incoming sediment from the water column. A peak in the reservoir abandonment index suggests that by the end of the 20th century, this floating vegetation mat had once again become well-established across the reservoir. This, in conjunction with the peak in the attenuation of land use component, suggests that this limited occupation of Koh Ker had, by the 20th century, perhaps begun to decline further and led to the city’s eventual abandonment.

### Combined record for Koh Ker

Overall, the occupation history of Koh Ker is more complicated and protracted than the epigraphic and architectural record would suggest. The palaeoecological record presented here strongly suggests that occupation of the city extended well beyond its ephemeral royal tenure during the reign of Jayavarman IV. The PTC3 record indicates that the construction of major monuments and the associated water infrastructure within the main temple precinct may have begun as early as the late 7th century C.E. Earthenware fragments and roof tiles found within the city and its surrounds provide context to suggest that occupation at Koh Ker extends back to the pre-Angkor period–at least to 500 C.E. [14, 87]. However it is common for Angkor-period urban sites to be constructed within or overlying proto-historic or pre-Angkor-period occupation sites (e.g. Prei Khmeng at Angkor, [88], and therefore archaeological context may provide limited support for such an early construction of Prasat Thom. It is, however, also possible that the moat had been excavated in association with a previous (and subsequently destroyed) structure or temple and had begun to accumulate sediment and autochthonous organic material decades or even centuries prior to the final completion and dedication of the replacement structure, Prasat Thom (much like the 8th century excavation of the moat at Bakong, at Haraiharalaya in Angkor, surrounding the 9th century temple and its 12th century central tower, see [23]).

The palaeoecological record has also revealed that, following the return of the seat of power to Angkor in 940–941 C.E., the occupation of Koh Ker likely persisted for at least the next seven centuries, and possibly beyond, at a reduced scale. There is evidence for fluctuating shifts in the intensity of land use throughout Koh Ker’s history, as well as a substantial decline in populations by the mid-15th century C.E. However, Koh Ker was not an ephemeral city established in virgin forest and utterly discarded two decades later, as has been previously asserted [3]. Instead, the city appears to have developed within an existing, smaller-scale Pre-Angkor-period site, which then continued to grow and function throughout the Angkor Period. This narrative supports the growing body of post-10th century archaeological evidence at the site, and importantly shows that the mobility of the royals and the elite may not translate to a similar mobility in the rest of the urban population, nor does it signify an agricultural collapse. While the brief tenure of Koh Ker as the capital city of the Khmer kingdom was, undoubtedly, politically significant, and the resulting elite influx would have engendered significant economic and demographic change, daily life may have been otherwise unaffected for the communities that had long been living and working in the area. It has been a recurring assumption in the traditional scholarship of the Khmer kingdom that a shift in the locus of power inevitably results in the wholesale migration of populations and the abandonment of urban centres (see [89]), with the 15th century abandonment of Angkor being the classic example. These results from Koh Ker can now complement the research from Angkor and other Khmer centres that have re-evaluated these assumptions and shown that residual populations often remain for decades or centuries beyond political abandonment (see [22, 23, 90, 91]).

## Supporting information

S1 Dataset (XLSX)
